# Professional accountability in a sternal bone marrow aspiration: a forensic case report

**DOI:** 10.11604/pamj.2020.36.255.21058

**Published:** 2020-08-06

**Authors:** Sami Bardaa, Narjes Karray, Zouhir Hammami, Samir Maatoug

**Affiliations:** 1 Department of Forensic Medicine, Habib Bourguiba University Hospital, Sfax, Tunisia

**Keywords:** Sternal bone marrow aspiration, aortic injury, pericardial tamponade, malpractice, liability

## Abstract

An aortic injury with concomitant pericardial tamponade caused by sternal bone marrow aspiration is rare. We report a case of fatal sternal bone marrow aspiration performed to a 73 year old man for the purpose of confirming the diagnosis of multiple myeloma. This puncture was followed by an injury in the aorta causing a pericardial tamponade and the death of the patient immediately after the aspiration. This paper stresses the precautions to be taken, by the operator, in certain particular situations that make the sternal bone marrow aspiration difficult and risky, and discusses the different types of operator´s liability that can be involved and their foundations.

## Introduction

Sternal bone marrow aspiration is an important medical procedure for the diagnosis of hematological, oncological and infectious diseases or for medical therapy monitoring [1]. It is often recommended through it is an invasive diagnostic method. As an invasive procedure, minor and major complications are described in literature. The minor complications are the local ones (bleeding and infection of puncture site). The major ones are caused by improper techniques or instrument usage. The fatal complications are due to ascendant aorta injury or the right ventricle one. Here, we report a case of fatal bone marrow aspiration following an aortic injury. The purpose of this paper is to remind the reader of the bone marrow aspiration technique, to highlight some precautions to be taken in particular situations such as the patient´s fragility. We discuss a possible liability of the operator in case of unexpected adverse effects following a sternal bone marrow aspiration.

## Patient and observation

**Figure 1:**
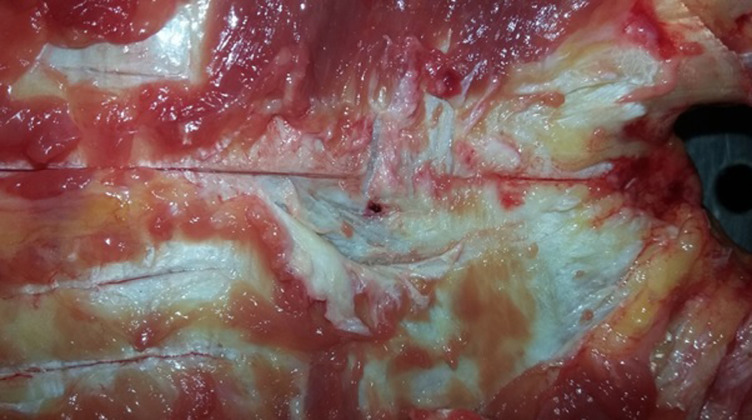
a crossing of the posterior table of the sternum by the trocar

**Figure 2:**
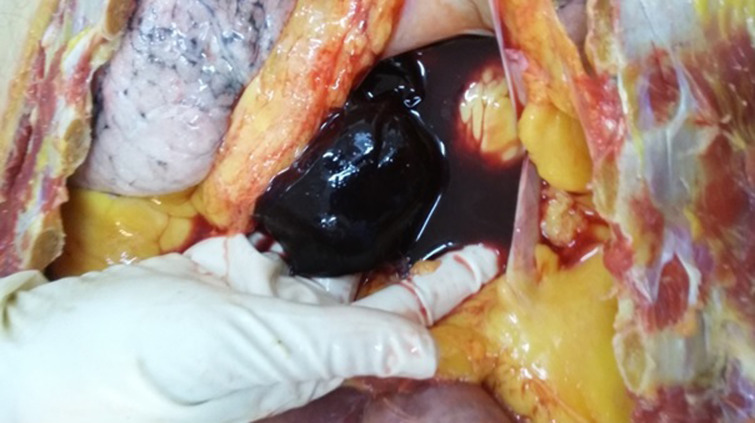
fluid and coagulated blood in the pericardial cavity

**Figure 3:**
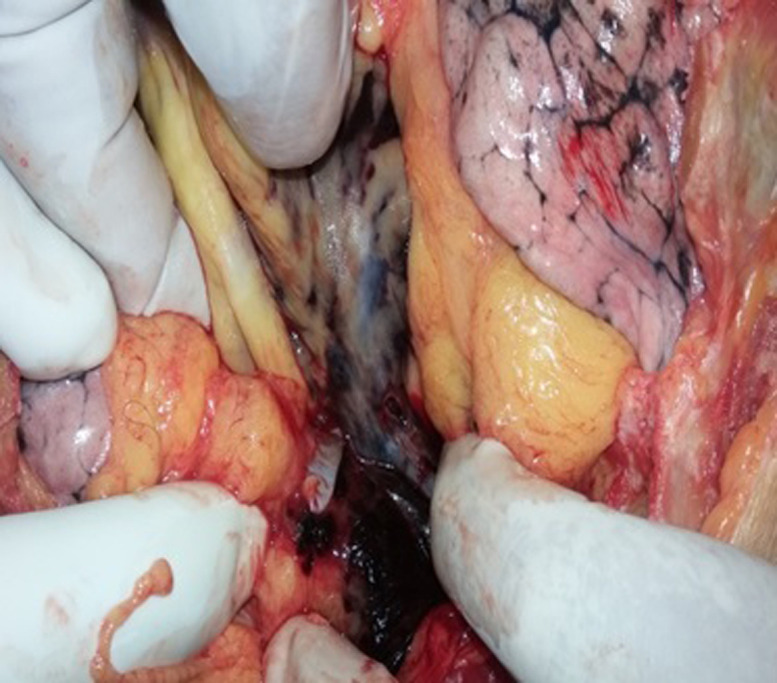
a perforating wound of the ascending part of the aorta

**Figure 4:**
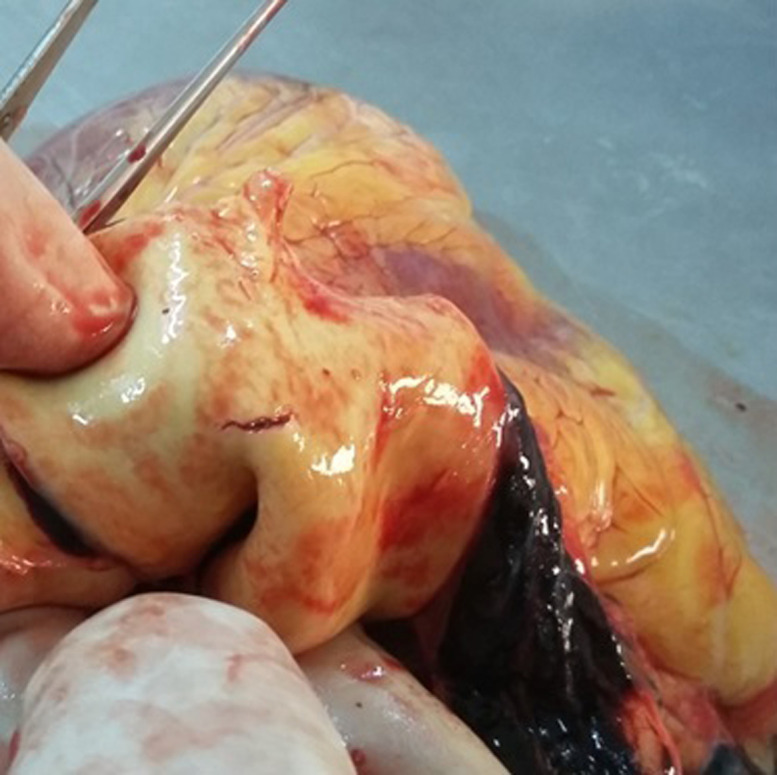
a wound on the inside of the aorta

A 73 year old man with a medical history of diabetes and under oral antidiabetics, had vertigo associated with important asthenia and cutaneous mucosa pallor. He was hospitalized for an etiological investigation. He had biological tests which revealed severe anemia with 3g/dl of hemoglobin and hyperproteinemia. The blood count showed a platelet count of 180,000. Diagnosis of multiple myeloma was suspected and sternal bone marrow aspiration was indicated to confirm the diagnosis. The technique was indicated because the iliac crests were fragile and thin. The sternal bone marrow aspiration was performed after having previously eliminated the contraindication. This procedure was performed according to the usual protocol. When the needle was introduced, the doctor noticed bleeding. Immediately, he removed the needle. Few seconds later, the patient who was still lying on the table, complained of pain, developed bradycardia, hypotension and a transient loss of consciousness. He was resuscitated without success and died within 40 minutes. His death required a medico-legal investigation, for which a forensic autopsy was conducted. At the external investigation, we observed a wound surrounded by a bruising in the anterior face of the sternum in front of the third intercostal space. No traumatic lesions were noted. At the autopsy, the examination of the sternal intern table noted a wound with a bruising (Figure 1). In addition to this wound, there was an area of bruising over the pericardium all around a perforated wound. The pericardial sac contained approximately 300ml of blood fluid and clot (Figure 2). Also, there was a perforating wound in the intra pericardial portion of the ascending thoracic aorta measuring 2 mm of diameter (Figure 3, Figure 4). The rest of the autopsy was normal.

## Discussion

Biological tests do not always provide enough information for the diagnosis of hematologic malignancies and infectious diseases. In some cases, a bone marrow examination is required for confirmation of a suspected clinical diagnosis or for monitoring medical therapy. It will be necessary for the confirmation of this diagnosis [2]. Aspiration may be done at several sites, but the sternum is supposed to be the easiest site from which to obtain the most cellular marrow [2]. Sternal bone marrow aspiration must be done according to a well-defined technique. This technique consists in practicing a puncture in front of the sternal anterior table with a special needle (a needle with sternal guard) while respecting the rules of asepsis. The site is chosen just to one side of the midline, at the level of the second or third intercostal space [2]. The thickness of the subcutaneous is determined during the induction of local anesthesia. The guard should be adjusted so that only a further 5mm advancement is possible [3]. The needle is held perpendicular to the bone, pushed through the periosteum of the sternum with clockwise-counterclockwise action until sensation of decreasing of the resistance. It is an index of entry in marrow cavity. Further advancement of the needle is avoided in order to prevent the achievement of the sternal posterior table [4].

The stylet is slowly removed, and the plunger is pulled back to aspirate approximately 0.5ml of bone marrow [4]. It is usually performed by an experienced practitioner [5, 6]. He should take precautions to avoid the occurrence of serious and even fatal complications. Thus, knowledge of the patient, its indication and the contraindications are required. There are few absolute contraindications to this method like hemophilia and severe disorders of coagulation. The relative contraindications are thrombocytopenia, infection of the skin at the point of puncture, a history of radiation therapy focused on the skin and finally any pathology leading to a change in normal anatomic landmarks [5, 6]. An X-ray of the chest prior to this procedure could show if there is an abnormality of the sternal bone as a contraindication or an additional reason for special precautions. In the reported case, sternal bone marrow aspiration was justified and there were no contraindications. In the case under study, an aortic injury with pericardial tamponade happened during the aspiration. Earlier, heart injury with pericardial tamponade due to sternal bone marrow aspiration has been rarely described in literature [7].

However, recently, occasional case reports of sternal puncture heart injuries have been published [7].They are very rare (approximately 0.8% of all sternal biopsies performed) [7]. Yet, the perforation of the anterior wall of the right ventricle is the anatomopathological lesion dominant in the literature [8, 9]. These aortic injuries can be explained by a technical error, a feature directly related to the patient or his illness or a lack of experience of the doctor performing the aspiration [8, 9]. In this situation, the operator can be sued for having involuntarily violated the bodily integrity of others and causing death in this case. Criminal medical liability is the heaviest and most prejudicial for the doctor. It is based on malpractice. In this case the operator can be prosecuted on the basis of articles 217 and 225 of the Tunisian Penal Code [10]. The bright red blood springing from the sternum when introducing the trocar should cause a sufficiently experienced doctor to realize a large vessel or heart has been injured. Failure to remove the trocar can lead to spontaneous hemostasis stop or at least delay rapid progression until the patient is treated by a specialist in cardiovascular and thoracic surgery. The potential liabilities for the damage caused and the lawsuits involve the administration, the department, the disciplines, the procedures and deserve attention.

## Conclusion

Sternal bone marrow aspiration is an invasive medical procedure. It is sometimes the only alternative to establish a diagnosis or to monitor the effectiveness of a treatment. Through this aspiration is presumed to be a simple act, it is practiced near the heart and large vessels and as a result, it sometimes exposes the patient to serious injuries. Injuries followed by the death of the patient are rare. To prevent them, an investigation before the practice of the puncture must be made in search of a contraindication including an abnormality of hemostasis and severe thrombocytopenia. A chest X-ray is also essential for finding a bone abnormality of the sternum. Also, a long experience is obligatory and the respect of the general rules of security are necessary. It should not be forgotten that in the event of a sternal puncture followed by a serious and unexpected incident, the victim may make the operator liable to the courts for involuntary damage to bodily integrity.
